# Lower Limb Deformity and Gait Deviations Among Adolescents and Adults With X-Linked Hypophosphatemia

**DOI:** 10.3389/fendo.2021.754084

**Published:** 2021-09-27

**Authors:** Gabriel T. Mindler, Andreas Kranzl, Alexandra Stauffer, Roland Kocijan, Rudolf Ganger, Christof Radler, Gabriele Haeusler, Adalbert Raimann

**Affiliations:** ^1^ Department of Pediatric Orthopaedics, Orthopaedic Hospital Speising, Vienna, Austria; ^2^ Vienna Bone and Growth Center, Vienna, Austria; ^3^ Laboratory for Gait and Movement Analysis, Orthopaedic Hospital Speising, Vienna, Austria; ^4^ Ludwig Boltzmann Institute of Osteology at Hanusch Hospital of OEGK and AUVA Trauma Center Meidling, 1st Medical Department, Hanusch Hospital, Vienna, Austria; ^5^ Medical Faculty of Bone Diseases, Sigmund Freud University, Vienna, Austria; ^6^ Department of Pediatrics and Adolescent Medicine, Division of Pediatric Pulmonology, Allergology and Endocrinology, Medical University of Vienna, Vienna, Austria

**Keywords:** XLH, gait analysis, gait deviations, hypophosphatemia, deformity, pseudofracture, enthesopathy, BMI

## Abstract

**Background:**

X-linked hypophosphatemia (XLH) is a rare genetic disorder characterized by lower limb deformity, gait and joint problems, and pain. Hence, quality of life is substantially impaired. This study aimed to assess lower limb deformity, specific radiographic changes, and gait deviations among adolescents and adults with XLH.

**Design:**

Data on laboratory examination and gait analysis results were analyzed retrospectively. Deformities, osteoarthritis, pseudofractures, and enthesopathies on lower limb radiographs were investigated. Gait analysis findings were compared between the XLH group and the control group comprising healthy adults.

**Patients and Controls:**

Radiographic outcomes were assessed retrospectively in 43 patients with XLH (28 female, 15 male). Gait analysis data was available in 29 patients with confirmed XLH and compared to a healthy reference cohort (n=76).

**Results:**

Patients with XLH had a lower gait quality compared to healthy controls (Gait deviation index GDI 65.9% +/- 16.2). About 48.3% of the study population presented with a greater lateral trunk lean, commonly referred to as waddling gait. A higher BMI and mechanical axis deviation of the lower limbs were associated with lower gait scores and greater lateral trunk lean. Patients with radiologic signs of enthesopathies had a lower GDI.

**Conclusions:**

This study showed for the first time that lower limb deformity, BMI, and typical features of XLH such as enthesopathies negatively affected gait quality among adolescents and adults with XLH.

## Introduction

X-linked hypophosphatemia (XLH, OMIM 307800) is a rare disorder of mineral metabolism associated with progressive rickets, profound deformities, and osteomalacia. Due to loss-of-function of mutation in the phosphate-regulating gene with homology to endopeptidases in the X chromosome (PHEX), dysregulation of fibroblast-like growth factor 23 (FGF23) leads to chronic renal phosphate wasting and impaired activation of 1,25 dihydroxyvitamin D [1,25(OH)2D] (1).

Patients with XLH can develop rachitic deformities of the lower limbs and short stature due to chronic phosphate depletion and associated growth plate pathologies. Musculoskeletal pain, dental abscesses, and fatigue further substantially impair quality of life (QoL) among patients with XLH (2). Therefore, patients of all ages with XLH should be treated in a multidisciplinary setting (3, 4).

Most patients with XLH report gait and joint problems, which are burdensome (5, 6). Thus, multiple surgical corrections of XLH-associated long bone deformities with bowing and maltorsion of the lower limbs are frequently required (7, 8).

Gait abnormalities have been a common feature among adults with XLH (6). In children with XLH who had no prior surgical interventions, a detailed analysis showed femoral and tibial malrotation and maltorsion, varus and valgus deformity of the lower limbs, and compensation mechanisms during gait (9).

Gait characteristics in adults have so far only been reported in a single study that only included a heterogenous group of nine non-surgically and surgically treated adults with XLH (10). However, a detailed and standardized analysis of lower limb deformity was not performed in this study.

Furthermore, a previous study has reported the radiographic features of adults with XLH (11). Moreover, some authors have shown a high incidence of lower limb deformities (12, 13). However, a comprehensive analysis of lower limb deformity and gait was not performed.

Thus, the current study aimed to analyze lower limb deformity, specific radiographic changes, and gait deviations among adolescents and adults with XLH who received prior surgical interventions and those who did not. Moreover, factors influencing gait quality were identified.

## Patients and Methods

This was a single-center study of adolescent and adult patients with XLH with and without previous surgical intervention performed at Orthopaedic Hospital Speising, Vienna. Data on patients with XLH aged older than 16 years were retrospectively analyzed using the dataset of our gait laboratory registry Furthermore, participants were actively recruited *via* the national XLH alliance “Phosphatdiabetes Austria”. The study was approved by the local ethics committee (EK37/2020).

### Inclusion/Exclusion Criteria

The inclusion criteria included patients aged above 16 years who have a genetically verified XLH and who presented with fused growth plates of the lower limbs at the time of examination. The exclusion criteria were patients with other types of hypophosphatemia and those with incomplete radiographic data.

### Data Acquisition

Laboratory examination and gait analysis results from maximum 6 months before and after radiographic examination were included in the analysis.

Full length anteroposterior (ap) radiographs (hip to ankle) of both lower limbs in standing position with centralized patella and with a calibration ball were obtained to analyze frontal lower limb deformity.

**Figure 1:**
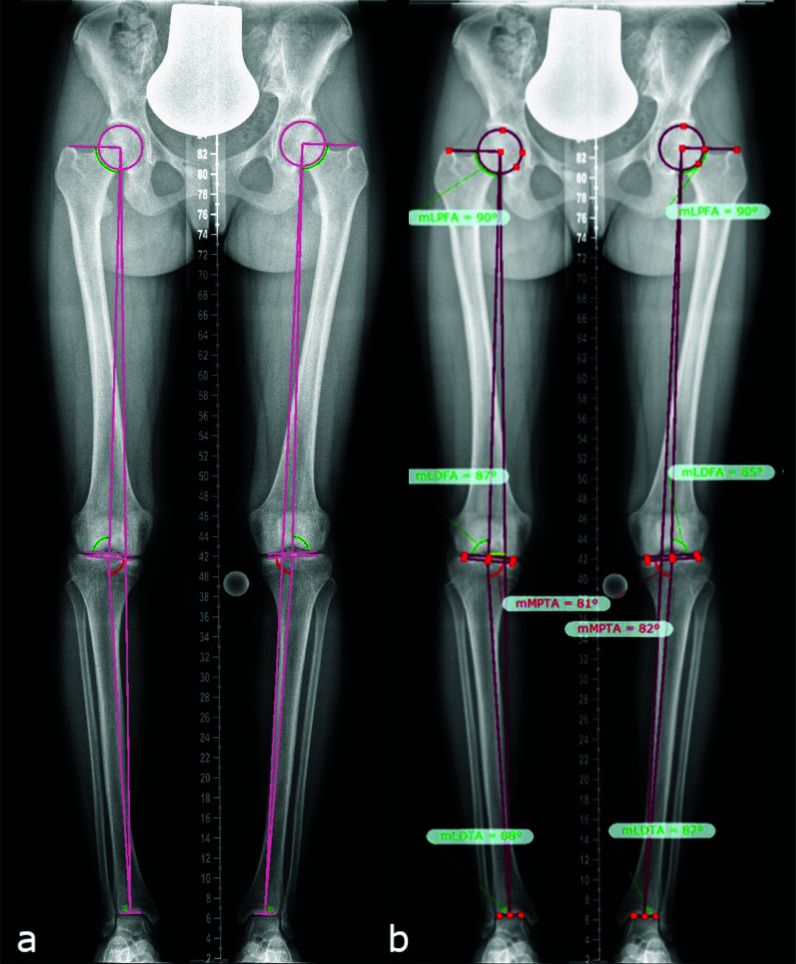
Lower limb deformity measurements on full-length anteroposterior radiograph images (hip to ankle) of both lower limbs in standing position **(A)**, as assessed using the TraumaCAD software according to standard measurement protocols **(B)** (14, 15), in a patient with mild XLH (18 y/o, female, BMI of 21.6 kg/m^2^, GDI of 89.8, no surgeries, receiving conventional treatment since childhood, and mild bilateral varus deformity proximal tibia).

Deformity analysis of ap radiograph images (hip to ankle) was performed by one examiner (A.S.) using the TraumaCad software (Brainlab AG, Munich, Germany) according to standard measurements (14, 15) (**Figure 1**). Long leg films were standardized with a centralized patella to minimize rotational effects.

Mechanical axis deviation (MAD), leg length discrepancy (LLD), mechanical lateral proximal femoral angle (mLPFA), mechanical lateral distal femoral angle (mLDFA), mechanical medial proximal tibial angle (mMPTA), and mechanical lateral distal tibial angle (mLDTA) were assessed in ap radiographs. On lateral radiograph, the femoral and tibial diaphyseal procurvatum angles were analyzed. Radiographic angles were compared with published values (14). Pseudofractures and enthesopathies of the lower limb were evaluated. Furthermore, radiographic scores were used to describe osteoarthritis of the hip (16), knee (17), and ankle (18).

### Gait Analysis

The modified Cleveland model for movement of the lower extremity and the Plug in Gait model for movement of the upper extremity marker set were applied. The Vicon motion capture system (Vicon, Oxford, the United Kingdom) (19, 20) was used for gait analysis.

To obtain kinematic data from a minimum of five force plate (AMTI Advanced Mechanical Technology Inc., Watertown, Massachusetts) strikes per foot, patients were instructed to walk a 12-meter walkway at a self-selected speed. A custom Matlab script (The MathWorks, Natick, Massachusetts, Version 2019a) was used to graph and compare data between groups. The Gait Deviation Index (GDI) was calculated according to the study of Schwartz and Rozumalski (21). A historic gait laboratory cohort of healthy adults was used.

Internal foot progression during the single support phase was referred to as intoeing. A lower range of motion (ROM) of the hip, knee, and ankle, as well as external rotation of the hip and knee were defined as values exceeding two standard deviations from that of the control group. A higher lateral trunk lean was defined as an increase in the medio-lateral ROM of the thorax exceeding two standard deviations from that of the control group.

### Statistics

The Kolmogorov–Smirnov test for testing normal distribution, independent *t*-test (data with normal distribution), and Mann–Whitney U test (data with non-normal distribution) were used to perform statistical analysis for the comparison of gait parameters between patients with XLH and age-matched controls. Statistical parametric mapping was used to assess waveforms (22). The statistical value of gait parameters was assessed using Matlab and SPM in Python (Statistical Parametric Mapping. Retrieved from www.spm1d.org). The least squares approach was utilized to quantify the strength of the relationship between BMI and GDI or lateral trunk lean in the linear regression models. Strength was evaluated using R², and the F-test was applied to evaluate statistical significance. For parameters with normal and non-normal distribution, Pearson’s or Spearman’s correlations were calculated, respectively. Data with normal distribution were assessed using the Shapiro–Wilk test. A p value of ≤ 0.05 was considered statistically significant. Data were processed using Jamovi version 1.1.19 (The Jamovi Project, 2019. Retrieved from https://www.jamovi.org; R Core Team 2018. R: Retrieved from https://cran.r-project.org/).

## Results

### Study Population

During the study period from January 2010 to April 2021, data on 51 adolescents and adults with XLH were eligible from the database. Eight patients were excluded due to incomplete radiographic data. Finally, 43 adolescents and adults with XLH (86 legs; 28 women, 15 men; mean age: 29 years) who underwent complete radiographic examination and 29 patients (20 women, 9 men; mean age: 32 years) with gait analysis data were included in this study.

The gait analysis data between the XLH group (n = 29, 58 limbs) and the control group (n = 76 healthy adults, 152 limbs) were compared. The gait parameters of patients with XLH treated non-surgically (n = 7) and those treated surgically (n = 22) were further analyzed in subgroups. The control group included 37 men and 39 women, with a mean age of 28.3 (range: 21–50) years, and data were obtained from our gait analysis database. The average weight of the control group was 69.0 (range: 47.4–101.7) kg; average height, 174.3cm (range: 155–198.0); and average BMI, 22.6 (17.6–29.1) kg/m^2^.

In total, 4 (16%) of 25 patients with available medical records received conventional medical treatment during childhood and at the time of radiographic examination. Meanwhile, 11 (42%) of 26 patients with available medical records did not receive any pharmacologic treatment during childhood. One patient was treated with burosumab, a monoclonal antibody to FGF23, starting at 3 weeks prior to gait analysis.

In total, 35 (81%) of 43 patients underwent limb alignment surgeries prior to this study. 23 patients (53%) had more than five multiple bony surgeries using guided growth, osteotomies, hexapod fixators, or intramedullary nails. Two patients (aged 60 and 71 years) had three arthroplasties (1 hip, 2 knees) at the time of examination.

Mean height of XLH patients (n = 40) was reduced and BMI elevated (**Table 1**), in particular 16 (40%) patients were < 150 cm tall. Moreover, 26 (65%) patients had an increased BMI, with 14 (35%) overweight (BMI: > 25 kg/m^2^) and 12 (30%), obese (BMI: > 30 kg/m^2^) classification.

**Table 1:** Demographic data of the study population including mean age, weight, height, and BMI and ranges (min–max) inside the brackets.

Study population (n = 43 patients)	Female	n = 28
	Male	n = 15
	**Age**	29 (16–71) years
	**Weight**	66.5 (46.9–96) kg
	**Height**	153.2 (138–167) cm
	**BMI**	28.6 (18.4–43.8) kg/m^2^
**Radiographic analysis**	**Valgus (MAD > 15 mm)**	21 (15–25) mm
		n = 6 legs
	**Varus (MAD > 10 mm)**	50 (13–164) mm
		n = 39 legs
	**JLCA**	2° (0°–10°)
		n = 86/86 legs
	**mLPFA**	100.2° (68°–123°)
		n = 86/86 legs
	**mLDFA**	94.1° (79°–122°)
		n = 86/86 legs
	**mMPTA**	84.9° (30°–98°)
		n = 86/86 legs
	**mLDTA**	90.5° (31°–114°)
		n = 86/86 legs
	**LLD**	9.4 mm (0°–52°)
		n = 41/43 patients
	**Femoral bowing**	18.9° (-5° to 83°)
		n = 48/86 legs
	**Tibial bowing**	6.7° (-9° to 39°)
		n = 45/86 legs
**Laboratory values (n = 23 patients)**	**Calcium**	2.37 (SD: 0.14) mmol/L
	**Phosphate**	0.68 (SD: 0.21) mmol/L
	**ALP**	125.85 (SD: 47.40) U/L
	**PTH**	80.42 (SD: 45.60) ng/L
	**25OH-Vitamin D**	50.92 (SD: 28.51) nmol/L

Radiographic analysis of mean angles measured using the TraumaCad software (Brainlab AG, Munich, Germany) according to standard protocols (15), mean femoral and tibial bowing angle, and mean lower limb discrepancy. Cases of varus and valgus, defined as MAD of > 15 mm and > 10 mm, respectively, are listed above. Number of legs and patients, marked with *. The mean laboratory values are listed above.

### Radiographic Findings of the Lower Limbs

Adolescents and adults with XLH had different types and degrees of lower limb deformity. Valgus and varus deformities were observed at varying levels (hip, knee, and ankle) (**Table 2**).

**Table 2:** Radiographic findings of patients with XLH who presented with varus and valgus deformity at the hip, knee, and ankle.

Deformity analysis	Varus	Normal	Valgus
*n = 86 legs*			
Hip (LPFA: 85–90)	88.4% (76)	8.1% (7)	3.5% (3)
Knee/femur (LDFA: 85–90)	52.3% (45)	40.7% (35)	7.0% (6)
Knee/tibia (MPTA: 85–90)	32.5% (28)	51.2% (44)	16.3% (14)
Ankle (LDTA: 86–92)	33.7% (29)	51.2% (44)	15.1% (13)

Percentages are listed inside the brackets. The standard angle measurements according to Paley et al. (14, 15) were used to analyze the full-length anteroposterior radiographs of both legs in standing position using the TraumaCad software (Brainlab AG, Munich, Germany).

The radiographic outcomes (n = 43 patients, 86 limbs) were frontal lower limb malalignment (genu varum: MAD, > 15 mm medial; genu valgum: MAD, > 10 mm lateral) in 52.3% (n = 45) of limbs (mean: 50 mm medial to 21 mm lateral). Knee valgus deformity was detected in 6 (7.0%) and knee varus deformity in 39 (45.3%) of 86 limbs. In total, 76 (88.4%) of 86 hips had proximal femoral varus deformity (mLPFA: > 90°). Lateral radiograph showed a mean anterior femoral bowing angle of 18.9° (n = 48 femora) and tibial bowing angle of 6.7° (n = 45). Patients without prior surgery (n = 7) presented with mild to severe frontal axis deviation (MAD = 25 mm lateral/valgus to 164 mm medial/varus). In total, 13 (30.2%) patients aged 17–71 years presented with pseudofractures (25 in 19 femora, 9 in 8 tibiae, and 5 in 4 fibulae). Among them, nine (69.2%) were aged < 30 years.

The radiographic scores indicated osteoarthritis in 68 (79.1%) of 86 hips, 56 (65.1%) of 86 knees, and 45 (52.3%) of 86 ankles. Moreover, 27 (69.2%) of 39 feet presented with degenerative changes in the talonavicular joint. In total, 35 (81.4%) patients had radiographic signs of enthesopathies, mainly at the lesser (n = 28/86 limbs) and the greater (n = 27/86 limbs) trochanter.

### Gait Deviations

Patients with XLH had a lower walking speed (p < 0.001) and broader step width (p < 0.001). Adolescents and adults with XLH had a low gait quality based on GDI, which is a global measure of gait quality (Mean 65.9%, SD 16.2%, range 27.6 - 92.2).

**Figure 2:**
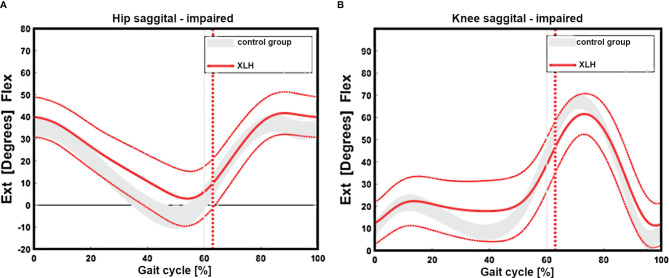
Gait cycle analysis showing overall impaired sagittal range of motion of the hip (n = 29 patients) (reduced extension, **(A)**. Reduced extension during stance and flexion during swinging of the group with XLH (**B**, red line + 1 SD) *versus* the control group grey curved area.

Regarding the sagittal profile, patients with XLH presented with a low hip, knee, and ankle ROM (**Figures 2A, B**). The main decrease in the sagittal ROM was observed in 27 (46.6%) of 58 knees (reference value = norm + 2 SD = 54.7°), 13 (22.4%) of 58 ankles (reference value = norm + 2 SD = 23.3°), and 12 (20.7%) of 58 hips (reference value = norm + 2SD = 35.8°).

**Figure 3:**
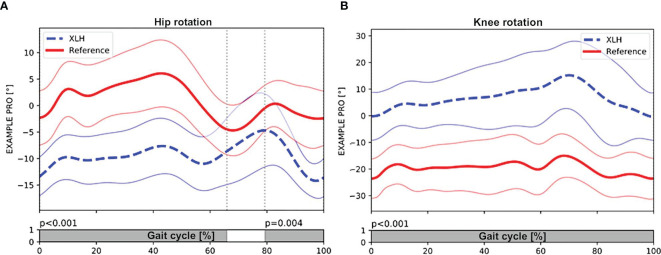
Patients with XLH (n = 29 patients) (dotted blue line, + 1SD) had significant external rotation of the hip during stance (p < 0.001) and swing phase (p = 0.004) compared with the control group (solid red line, + 1 SD) **(A)**. **(B)** also showing significant internal rotation of the knee during the whole gait cycle (p < 0.001). Gray bar indicates the level of significance (p < 0.005) between the group with XLH and the control group during the gait cycle. Meanwhile, the white bar indicated non-significance (p > 0.005).

Gait analysis showed a significantly greater external femoral rotation as well as a lesser tibial external rotation in the XLH group (p < 0.001, **Figures 3A, B**). The surgically treated group had lower rotation deviations of the femur (p = 0.001) and the tibia (p = 0.001) than the non-surgically treated group. In total, 15 (51.7%) of 29 patients with XLH (6 of 9 who did not undergo surgery) presented with intoeing.

**Figure 4:**
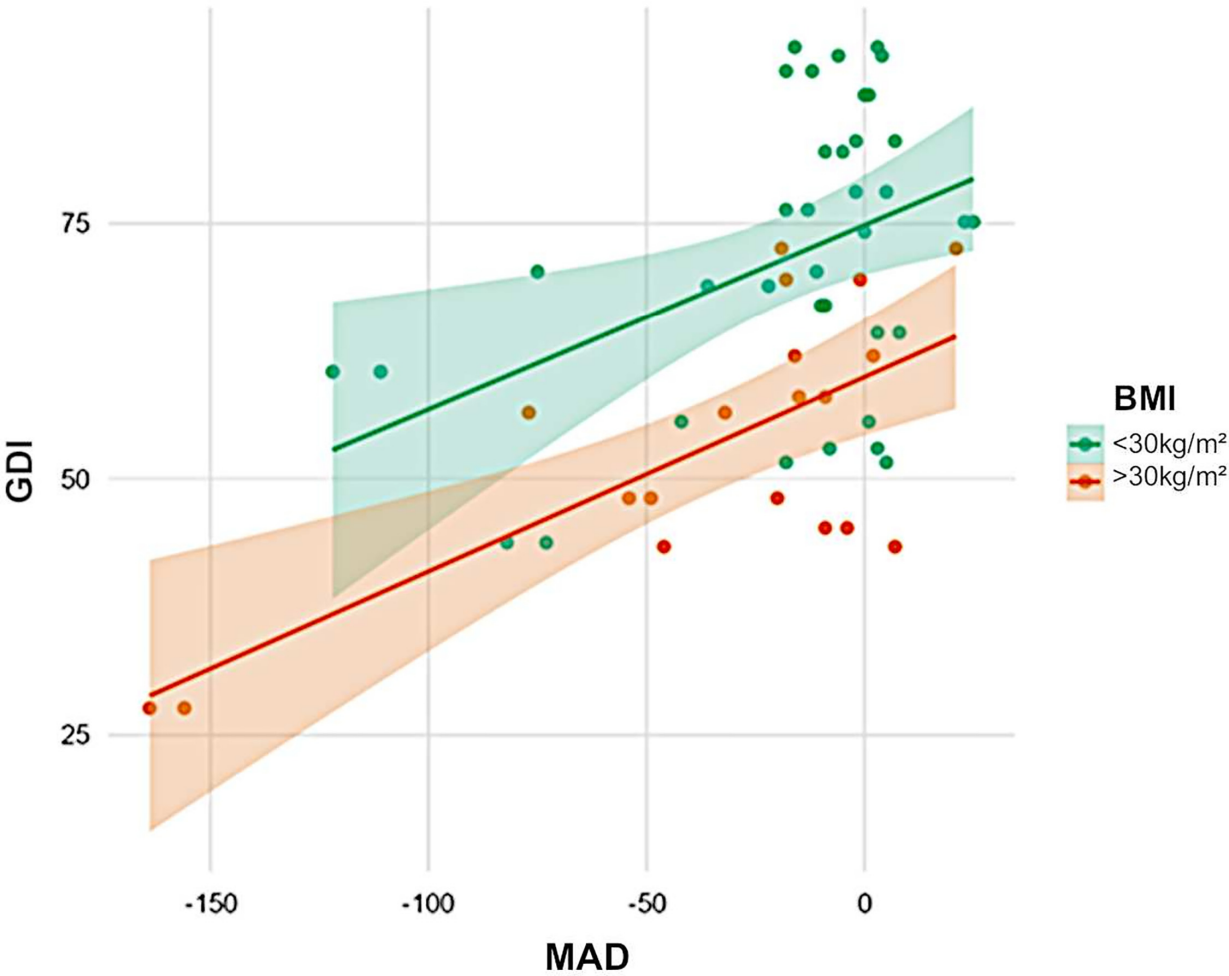
Influence of weight and axial deviation on gait scores. Obesity (orange) was associated with low GDI scores (green). Furthermore, varus was negatively correlated with GDI. Obese patients with varus deformity had the lowest scores.

In total, 14 (48.3%) of 29 patients had a greater lateral trunk lean (waddling gait), defined as a frontal thorax ROM of > 7°. Patients with a higher BMI (R² = 0.41, p < 0.001) and those with varus knee deformity (p = 0.002, **Figure 4**) presented with a greater lateral trunk leaning. The proximal femoral hip angle (mLPFA) was not correlated with lateral trunk lean (R² = 0.17, p = 0.241). Furthermore, there was no association between thorax length in relation to leg length and lateral trunk lean (p > 0.05).

The tibial anterior bowing angle (procurvatum) was associated with a lower knee and ankle ROM (n=42 limbs, knee: R² = 0.37, p < 0.001; ankle: R² = 0.21, p = 0.011) and thus, gait quality (n = 21 patients, R² = 0.27, p = 0.004). However, the femoral anterior bowing angle (procurvatum) did not influence hip/knee motion or GDI significantly (n = 32, p = 0.869).

**Figure 5:**
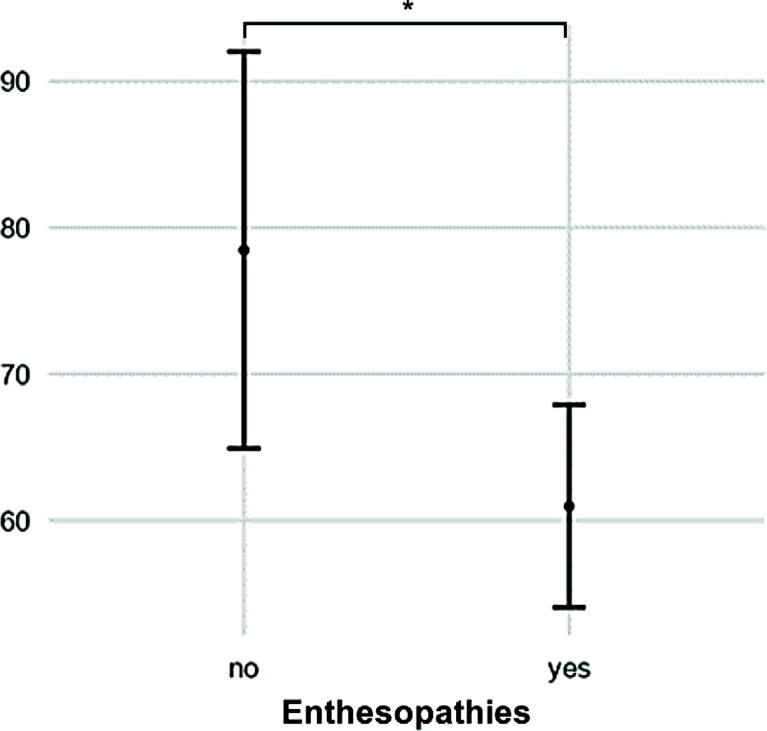
Patients with radiographic signs of enthesopathies had low GDI scores compared with those without (p = 0.022). (* = p > 0.05).

Patients with enthesopathies on anteroposterior radiographs of the lower limb had a low gait quality based on GDI (p = 0.022, **Figure 5**). A high BMI was associated with a low gait score (GDI: R² = 0.32, p = 0.002) and a greater lateral trunk lean (R² = 0.17, p = 0.002).

Standard laboratory parameters (n = 23) including serum calcium (Ca), phosphate, total alkaline phosphatase, and 25-hydroxy vitamin D [25(OH)D] levels were not associated with GDI. Patients with a high BMI had a low 25(OH)D level. However, there was no difference in Ca or PTH levels.

## Discussion

The current study showed that adolescents and adults with XLH presented with a significantly low gait quality based on GDI as well as lateral trunk lean (“waddling”). Common features were complex lower limb deformities and signs of osteoarthritis, pseudofractures, and enthesopathy.

Lower limb deformity and gait impairment can substantially reduce QoL among adults with XLH (6). Aside from pain, fear of falling is common among adults with XLH, thereby resulting in reduced participation in social and public activities (23) and contributing to the burden of disease. Therefore, further studies that aim to not only identify and quantify contributing factors but also improve the multidisciplinary management of patients with XLH are warranted (6).

Lower limb alignment is important in biomechanical function, thus a normal gait. The characterization of biomechanical alterations in the lower limb among patients with XLH represents an essential prerequisite to assess the effects of medical or nonmedical interventions on factors contributing to burden of disease (24).

Although there are studies showing extremely high rates of lower limb deformity (12, 13, 25), there is currently no detailed measurement data regarding lower limb deformities among adults with XLH. Therefore, lower limb deformity among adults with XLH cannot be described and quantified. Zhang et al. (12) revealed that about 95.9% of patients with XLH (n = 217) presented with lower limb deformities. These were categorized as genu varum, genu valgum, and complex deformity. In XLH, lower limb deformity was defined as a complex deformity caused by additional anteroposterior (procurvatum) or torsional deformity, which was not described in previous studies (12, 13).

**Figure 6:**
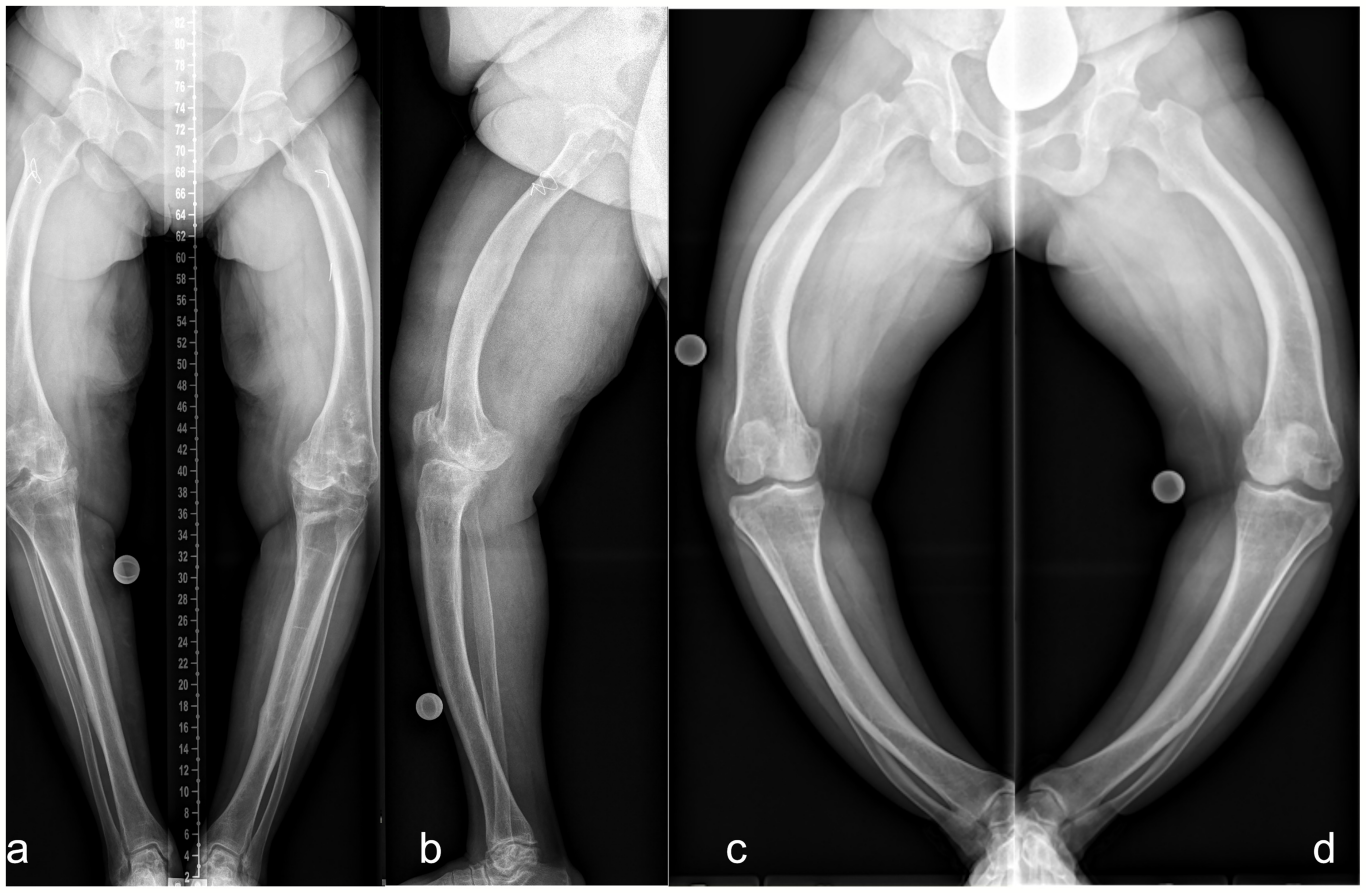
Lower limb deformity of patients prior to surgical frontal plane correction at our hospital. Left (**A** frontal, **B** sagittal): 50 y/o, female, five prior surgeries, GDI: 43.7, BMI: 28.7 kg/m^2^, conventional oral medication. Right: 33 y/o, female, no previous surgeries prior to presentation, GDI: 27.6, BMI: 37.6 kg/m^2^. Full-length anteroposterior radiograph (hip to ankle) of both lower limbs in standing position was not performed due to severe varus deformity. Thus, radiograph image of each leg was taken individually **(C, D)**.

**Figure 7:**
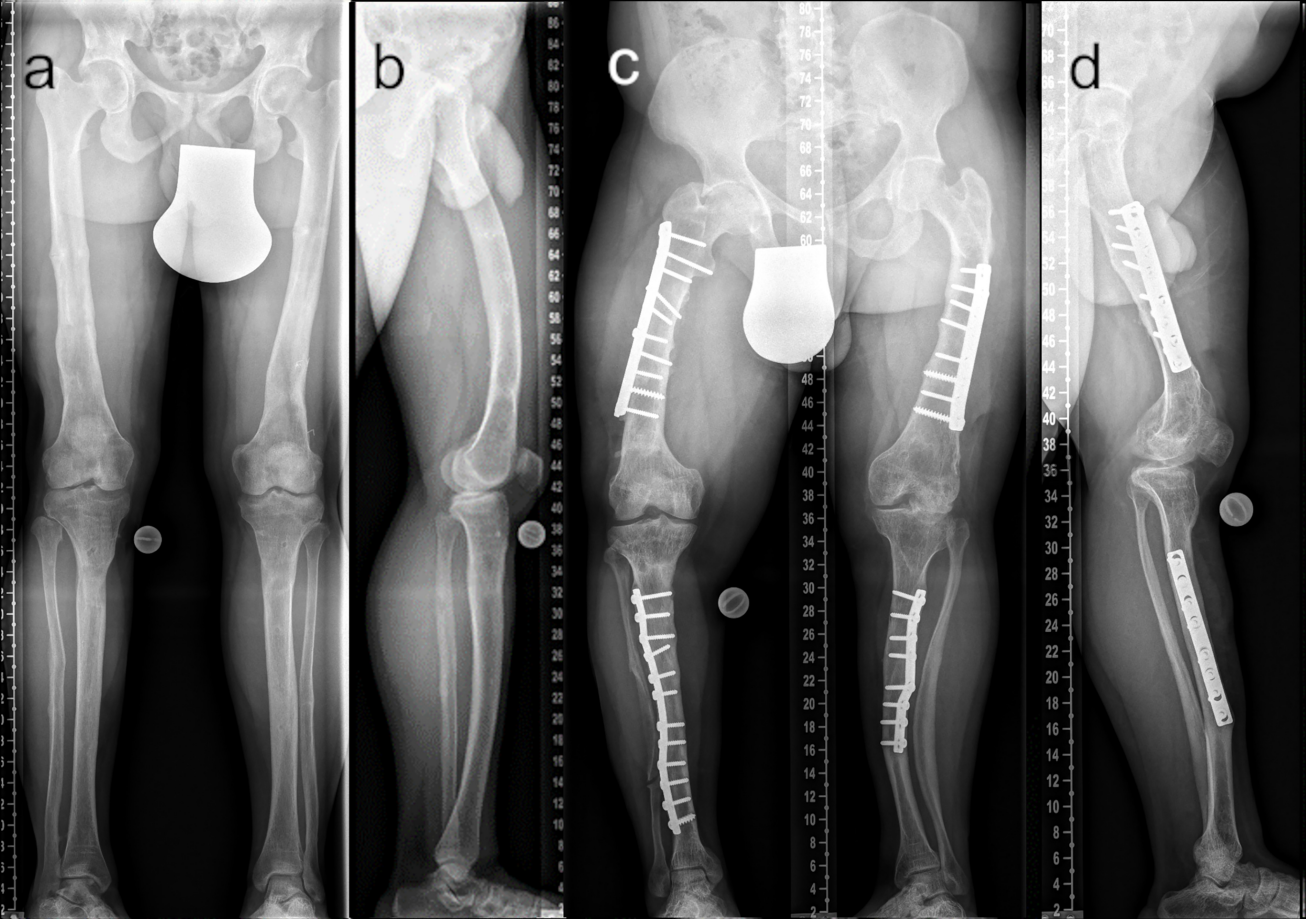
Comparison of adequate (**A** ap; **B** lateral) and insufficient (**C** ap; **D** lateral) surgical frontal and sagittal plane correction. The patient depicted in **(A, B)** underwent multilevel lower limb correction at our hospital (28 y/o, male, GDI: 62.0, BMI: 38.3 kg/m^2^, conventional oral medication). The patient depicted in **(C, D)** presented with multiple prior surgeries performed elsewhere (37 y/o, male, GDI: 43.3, BMI: 33.3 kg/m^2^, conventional oral medication).

In our patient group, the severity of lower limb alignment deviations ranged from normal to severely pathologic (**Figures 6A–D** and **7A–D**). Compared to children with XLH who did not receive prior surgical intervention (9), adults had a lower incidence of valgus deformity and a higher incidence of varus deformity of the lower limb. However, similar rotational abnormalities with signs of a higher external femoral torsion and a lower external tibial torsion were observed during gait.

According to Horn et al. (7), patients who underwent frontal plane alignment correction and who had a neutral mechanical axis did not complain of significant residual torsional malalignment even though diaphyseal bowing was not completely corrected. Our data partly contradict these findings. Rotational deformity, including a high rate of intoeing, might have significantly contributed to gait deviations in adults with XLH, thereby causing burden of disease.

The pseudofracture rate (30%) in our study group was similar to that of other populations (13). Looser zones (pseudofractures) were found in adults with XLH, increasing with age It occurred in 28% of patients aged under 30 years. However, none of the patients aged under 18 years presented with pseudofractures (11). The youngest participant with pseudofractures on radiographic analysis was aged 17 years, and this patient underwent surgical intervention.

Anterior bowing of the tibia and femur (procurvatum), which is a sagittal plane deformity parameter, was not correlated with pseudofracture. Thus, this disease-specific radiographic finding may originate from other pathomechanisms compared with deformities and associated biomechanical strains only. Furthermore, our study did not show a correlation between the number of pseudofractures and gait quality. A previous study has shown the benefits of conventional treatment for the quality and quantity of bone mineralization among adult patients with XLH (26).

Enthesopathies and pseudofractures can significantly contribute to burden of disease (6). However, a reliable method for quantifying enthesopathies is yet to be developed (24). In this study, the negative influence of enthesopathies on gait quality confirmed the importance of this parameter among adults with XLH.

A murine model showed a higher occurrence of enthesopathies and joint structural alterations over time. Furthermore, all Hyp mice developed peripheral enthesopathies, thereby showing the mineralization of fibrochondrocytes at the achilles tendon and plantar fascia ligament insertions of the calcaneal tuberosity at the 12-month follow-up (27). Our study group showed comparable findings on detailed radiographic analysis. Thus, further research focusing on foot and ankle deformity among adults with XLH must be performed.

Enthesopathies may not respond adequately to conventional medical therapy among adults with XLH (28). Consequently, further research should be conducted to prevent a decrease in gait quality caused by this radiographic change.

The development of early and severe osteoarthritis is another highly relevant factor in the skeletal assessment of adults with XLH (6). Our cohort presented with a high rate of degenerative changes in the hip, knee, and ankle joints. However, only two patients underwent prior joint replacement surgeries.

Joint replacement has been effective in treating osteoarthritis among adults with XLH (29, 30). The surgical intervention was technically challenging due to severe deformity and poor bone quality (29).

Compared with the abovementioned studies, joint replacements in the current research occurred at a later age with consideration of the overall lower mean age of our study population. Joint replacement surgery may be postponed in patients with symptomatic osteoarthritis due to severe lower limb deformity. Hence, such a notion should be considered. In our study group, this finding was observed in at least three patients.

Steele et al. (10) comprehensively described the skeletal affection of the upper and lower extremities and spine in adults (n = 9). Degenerative changes in the entire skeletal apparatus were high. The gait characteristics (higher lateral trunk lean, lower hip extension and knee extension during stance) were similar to those reported in our study. However, an analysis of lower limb deformity was not performed, and there were no details regarding rotational and torsional deformities.

Lower limb alignment influences thorax movement among patients with XLH (9). Waddling is described as a disease-specific gait deviation in patients with XLH (9, 10, 31). However, this term has not been clearly defined, and its use might be inappropriate when considering patients’ demands in the context of the psychosocial aspects of burden of disease. The term waddling was replaced with increased lateral trunk lean and found similarly high rates in adults compared to children with XLH (9). The increased lateral trunk lean as the compensation mechanism of varus limb deformity could again be observed in adults similar to prior reports of children with XLH (9). We hypothesized that patients with a longer thorax (in relation to leg length) were more likely to have a greater lateral trunk lean during gait, which was not the case in our series. LPFA, which is an indicator of frontal hip deformity (varus/valgus), was not correlated with lateral thorax motion. However, it might influence hip abductor moment due to a change in lever arm. Therefore, gluteal insufficiency and proximal femoral varus deformity may not be the main causes of an increased lateral trunk lean among patients with XLH.

The current study first revealed the impact of a high BMI on gait quality among adults with XLH. Patients with a high BMI presented with lower gait scores and a higher incidence of increased lateral trunk lean. While the value of BMI measurement in populations with short stature is discussed controversially, these data contribute to characterize the role of metabolic control in XLH. In line, previous studies have shown a high prevalence of overweight in adult XLH populations (32) and the correlation between a high BMI and a lower gait quality among children with XLH (9).

Most adults with XLH had joint stiffness (5), which significantly contribute to disease burden. The current study showed a significant decrease in hip, knee, and ankle joint ROM during gait. Thus, stiffness is a clinically relevant and measurable parameter. Interestingly, procurvatum of the tibia (anterior bending) was associated with a decreased ankle and knee ROM and overall gait quality (gait deviation index). Thus, joint stiffness, which is a typical symptom of XLH, might be aggravated by sagittal plane deformity.

This study shows the importance of lower limb deformity correction in improving gait among patients with XLH. Femoral and tibial varus deformity, torsional deformity, and tibial procurvatum deformity and a higher BMI may contribute to a lower gait quality. Patients with prior alignment surgeries are more likely to present with lower rotational deviations. However, due to group heterogeneity, further comparison was not performed.

### Limitations

This study had several limitations. Included patients received regular medical care at multiple medical facilities across Austria and Europe, as reflected by extremely diverse pharmacological treatment regimens at the time of examination as well as extensive heterogeneous histories of medical treatment. Most patients were in the crucial phase of linear growth prior to the development of current treatment standards and multidisciplinary approaches for rare bone disorders. The surgically treated patients underwent various and multiple surgical procedures, thereby reflecting the high osteotomy rates within the last decades, which further contributed to group heterogeneity. Although the diversity of medical and surgical history reflects real-life clinical settings in XLH care, the outcomes of this study might be blurred by inhomogeneity. Nevertheless, the finding of several specific characteristics in this cohort underline the relevance and applicability of our outcomes in defiance of phenotypic and therapeutic variability in XLH.

Whilst symptoms and burden of disease were assessed retrospectively in this study, functional or QoL scoring was not available in the analysis. Complete (preoperative) lower limb deformity assessment includes lateral full leg radiography and torsional magnetic resonance imaging or computed tomography scan. However, these additional examinations were not accessible to all patients in this study. Gait analysis can examine the rotational and torsional components of lower limb deformity (9, 33). To detect the possible influence of spinal degeneration on gait, spine radiograph might be necessary. Furthermore, future studies of a spine motion marker should be conducted to assess spine movement among adult patients with XLH.

To minimize ionizing radiation on the gonads, all standardized long leg radiography images were obtained using a gonad shield based on the local standards of care. However, this protective measure inhibits the detection of changes in the sacroiliac joint and enthesopathies of the sacrospinous ligament.

Due to disease-specific maltorsion, lateral radiographic angles were challenging to measure in some cases; therefore, only the procurvatum shaft angle was evaluated.

## Conclusion

Adolescents and adults with XLH presented with an increased lateral trunk lean, lower gait scores, osteoarthritis, enthesopathies, and pseudofractures. This study for the first time showed that lower limb deformity (such as MAD, lateral bending, and malrotation), BMI, and features specific in XLH (such as enthesopathies) negatively affected gait quality among adolescents and adults with XLH. Therefore, patients with XLH require a comprehensive and adequate treatment in a multidisciplinary setting. Assessment of lower limb deformities and gait deviations should be implemented in future diagnostic and therapeutic studies to address this important aspect of disease burden in XLH patients.

## Data Availability Statement

The raw data supporting the conclusions of this article will be made available by the authors, without undue reservation.

## Ethics Statement

The studies involving human participants were reviewed and approved by Ethikkommission der Wiener Krankenhäuser der Vinzenz Gruppe Gumpendorferstraße 108 1060 Wien ethikkommission.wien@vinzenzgruppe.at (EK37/2020). Written informed consent to participate in this study was provided by the participants’ legal guardian/next of kin.

## Author Contributions

GM, AK, AS, RK, AR, RG, CR, and GH contributed to conception and design of the study. GM and AS wrote first draft of manuscript. AR wrote sections of the manuscript. GM, AS, RK, GH, and AR did clinical data collection. AK and AR performed statistical analysis. AK collected gait data. GM, AK, AS, and AR performed data analysis. All authors contributed to the article and approved the submitted version.

## Conflict of Interest

AR and RK received non-related honoraria from Kyowa Kirin for consultancy and scientific presentations. RG and CR received non-related honoraria from Nuvasive Inc. and Smith and Nephew for consultancy. The authors declare that the research was conducted in the absence of any commercial or financial relationships that could be constructed as a potential conflict of interest.

## Publisher’s Note

All claims expressed in this article are solely those of the authors and do not necessarily represent those of their affiliated organizations, or those of the publisher, the editors and the reviewers. Any product that may be evaluated in this article, or claim that may be made by its manufacturer, is not guaranteed or endorsed by the publisher.
